# Impact of an Electronic App on Resident Responses to Simulated In-Flight Medical Emergencies: Randomized Controlled Trial

**DOI:** 10.2196/10955

**Published:** 2019-06-07

**Authors:** Nur-Ain Nadir, Courtney J Cook, Raymond E Bertino, Marc D Squillante, Cameron Taylor, David Dragoo, Gregory S Podolej, Jessica D Svendsen, Jessica L Fish, Jeremy S McGarvey, William F Bond

**Affiliations:** 1 Department of Emergency Medicine University of Illinois Peoria, OSF St Francis Medical Center Peoria, IL United States; 2 Jump Simulation, OSF Healthcare Peoria, IL United States; 3 Department of Radiology University of Illinois Peoria, OSF St Francis Medical Center Peoria, IL United States; 4 Ministry Analytics OSF Healthcare Peoria, IL United States

**Keywords:** in-flight medical emergencies, ground medical control, commercial aviation, simulation

## Abstract

**Background:**

Health care providers are often called to respond to in-flight medical emergencies, but lack familiarity with expected supplies, interventions, and ground medical control support.

**Objective:**

The objective of this study was to determine whether a mobile phone app (airRx) improves responses to simulated in-flight medical emergencies.

**Methods:**

This was a randomized study of volunteer, nonemergency resident physician participants who managed simulated in-flight medical emergencies with or without the app. Simulations took place in a mock-up cabin in the simulation center. Standardized participants played the patient, family member, and flight attendant roles. Live, nonblinded rating was used with occasional video review for data clarification. Participants participated in two simulated in-flight medical emergencies (shortness of breath and syncope) and were evaluated with checklists and global rating scales (GRS). Checklist item success rates, key critical action times, GRS, and pre-post simulation confidence in managing in-flight medical emergencies were compared.

**Results:**

There were 29 participants in each arm (app vs control; N=58) of the study. Mean percentages of completed checklist items for the app versus control groups were mean 56.1 (SD 10.3) versus mean 49.4 (SD 7.4) for shortness of breath (*P*=.001) and mean 58 (SD 8.1) versus mean 49.8 (SD 7.0) for syncope (*P*<.001). The GRS improved with the app for the syncope case (mean 3.14, SD 0.89 versus control mean 2.6, SD 0.97; *P*=.003), but not the shortness of breath case (mean 2.90, SD 0.97 versus control mean 2.81, SD 0.80; *P*=.43). For timed checklist items, the app group contacted ground support faster for both cases, but the control group was faster to complete vitals and basic exam. Both groups indicated higher confidence in their postsimulation surveys, but the app group demonstrated a greater increase in this measure.

**Conclusions:**

Use of the airRx app prompted some actions, but delayed others. Simulated performance and feedback suggest the app is a useful adjunct for managing in-flight medical emergencies.

## Introduction

Epidemiologic evidence for in-flight medical emergencies from a ground-based medical support system estimated that medical emergencies occur in 1 of every 604 flights [1]. This is likely an underestimate because no mandatory reporting system exists, and uncomplicated issues often go unreported [2]. Air travel is increasing, with 895.5 million passengers flying in 2015 [3], leading to an increased frequency of in-flight medical emergencies. In one study, 42% of 418 health care providers surveyed reported being called on to give aid in an in-flight medical emergency [4].

The Federal Aviation Administration mandates that US-based airlines carry basic first aid kits stocked with bandages and splints, and at least one automated external defibrillator must be available [5]. Beyond the basic kit, no national or international standards exist, although there have been recent calls for consistency [6,7].

Health care personnel are also unlikely to be familiar with medical kit contents, flight crew communication, and medical emergency protocols [4]. Clinicians’ expertise typically consists of their specialty training and life support courses. Emergency response training is often limited as emergency medicine is not a mandatory rotation in medical education [8]. Although helpful, ground-based medical consultation support services (ground medical control) still depend on volunteers to be their “eyes and ears” [1,9]. The assumption is that volunteers will find and report clinical information relevant to the presenting medical emergency [1,10].

Comfort attending to an in-flight medical emergency is likely to vary substantially across provider backgrounds. Thus, there is a need for education about the environment and scenario-based basic in-flight medical emergency response training. In recent months, the aviation and health care industries have recognized this and called for education in emergency stabilization and flight medicine at both graduate and undergraduate levels [11,12].

Although several authors have discussed the management of in-flight emergencies [13-18], little real-time decision support exists outside of ground medical control. Normal emergency response mobile phone apps or cognitive aids may not take the environment into account. In response to this perceived need, a mobile phone app was designed by emergency, aerospace medicine, and radiology physicians (airRx) [19] to assist licensed health care personnel in dealing with the most common in-flight medical emergencies. The app offers complaint-specific recommended actions, care algorithms, and in-the-moment information regarding the likely available medications. While serving as a real-time decision support reference, the app also provides a method of just-in-time training (JITT) [20]. Pertinently, the JITT approach has been successful in on-the-job training for first responders in unfamiliar situations [21]. Studies have also shown that mobile phone-based cognitive aids promote adherence to protocols in both real and simulated clinical scenarios [22-24]. Therefore, a JITT-based mobile phone cognitive aid or app is a reasonable approach to delivering focused learning during an in-flight medical emergency. The objective of this study was to determine the usefulness of the airRx mobile phone app in responding to simulated in-flight medical emergencies. Our secondary objective was to examine whether access to the airRx app would increase confidence to respond to an in-flight medical emergency.

## Methods

### Design

**Figure 1:**
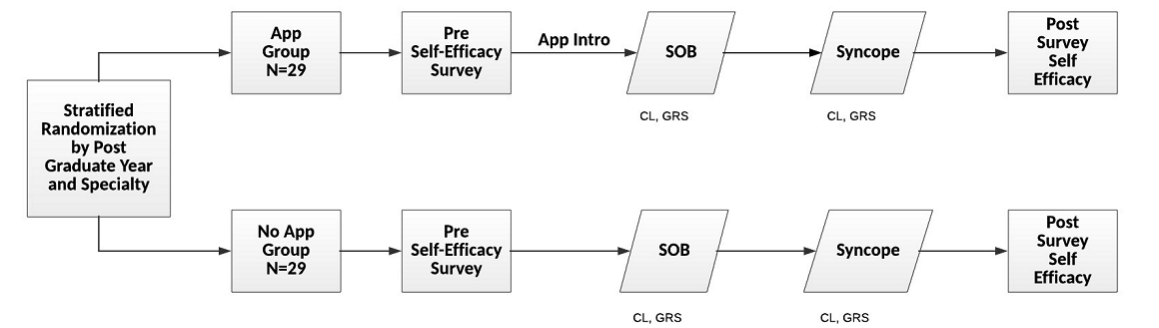
Study design. Participants were debriefed after the postsurvey. CL: checklist; GRS: Global Rating Scale; SOB: shortness of breath; SYN: syncope.

This was a prospective randomized controlled trial. Fifty-eight participants were block randomized by postgraduate year and specialty area to simulated in-flight medical emergencies with and without access to a smart device app. Although block randomization was used to assign participants to each group, the assessment and treatment of the two chief concerns—shortness of breath and syncope—were not randomized. Anticipating a case order effect, all simulations were run in the sequence order depicted in Figure 1 (overall study flow), with the shortness of breath case preceding the syncope case. This study design was approved by the University of Illinois–Peoria, Peoria, IL, Institutional Review Board.

### Participants

Participants were solicited from non-emergency medicine residency programs including diagnostic radiology, family medicine, internal medicine, pediatrics, psychiatry, combined medicine-pediatrics, and obstetrics and gynecology. Emergency medicine residents were excluded given their expertise and training in management of emergencies. Participants’ performances were kept confidential. They were compensated through a US $25 gift card and a copy of the airRx app at no cost to them. Participants were instructed to keep the scenarios confidential to minimize the relay of scenario information to future participants.

### Intervention

**Figure 2:**
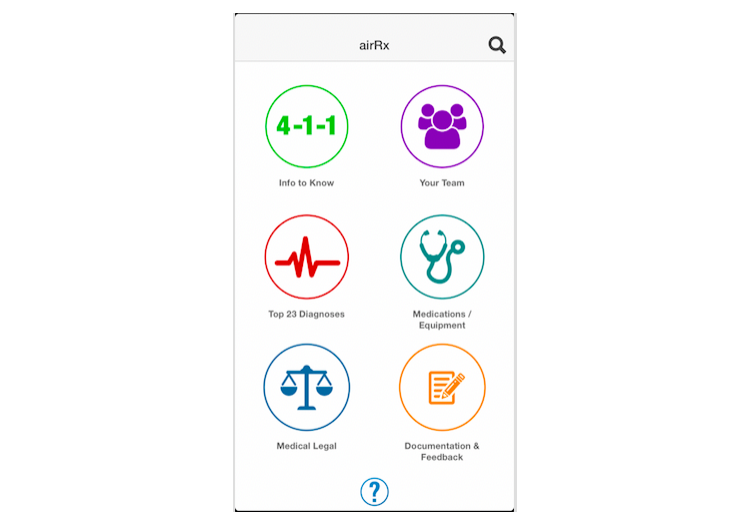
Screenshot of the airRx app.

The intervention tested is an app known as airRx (Figure 2). It was initially funded by a nonprofit organization and is now freely available on both the iOS and Android mobile phone platforms. It was created by the authors (RRB, MDS, CJC) and nonauthors (Joshua Timpe, MD; Claude Thibeault, MD; Paulo Alves; and John Vozenilek, MD) and designed to help non-critical care, non-emergency health care professionals manage common in-flight medical emergencies. The app version (airRx version 1.2.1, 2016) [19] was kept constant during the study. The app has a section on “universal starters” for users to consider for any in-flight medical emergency. There are also sections on medications and equipment to expect on most US major airlines, a complaint-based set of algorithms for management, and medicolegal information.

### Case Development

Syncope and shortness of breath were chosen as our in-flight medical emergency scenarios because these are the top two commonly occurring in-flight medical emergencies noted in the literature [1,12]. Case development followed standard simulation case creation guidelines [22]. Real flight attendants (United Airlines, Chicago, Illinois) also participated in the process of scenario design and pilot testing to ensure content validity. Cases are available in Multimedia Appendix 1.

### Simulation Environment

The study took place at a university hospital-affiliated simulation center. Space and movement limits that mimicked the floor distances of a Boeing 737 aircraft were created within a simulation laboratory with audiovisual recording capability.

The simulation center has a cadre of standardized participants who undergo general and scenario-specific orientation. The standardized participants went through dry runs of each scenario, received feedback on their performance, and were given earbuds for prompts in real time. In each scenario, there was one standardized participant passenger who became ill and one standardized participant passenger bystander who had relevant information if asked. Stable actor cohorts played these roles. Pathologic physical exam findings were given on cue through prewritten cards from the bystander standardized participant because healthy patient standardized participants could not mimic symptoms such as wheezing.

For each case there were also two standardized participant flight attendants who communicated with the investigators in the simulation control room (“pilot” and “ground medical support”) and relayed responses to the participants. Real flight attendants trained standardized participants to portray flight attendant roles through direct observations of their performance in pilot simulations, video review, and discussion of planned responses to questions. To isolate participant performances, we instructed the flight attendants to be helpful and follow directions but wait to inform ground medical control until instructed. Thus, we controlled for variable airline protocols, flight attendant training, and individual responses expected in real life.

All participants were prebriefed on the general premise of the simulation, safe space principles, and learner contracts, and given the opportunity to ask questions via a standardized script by the same personnel. Both the control and intervention groups were allowed to use any other phone apps they had on their personal smart device that would be accessible during airplane mode. The app group had up to 15 minutes to familiarize themselves with the app.

Scenarios began with the participants sitting in the simulated cabin with a brief pause before flight attendants announced the in-flight medical emergency and called for assistance; these were run for 8 minutes. The length of the simulation was determined based on pilot simulation cases in which, on average, most critical actions were completed by participants by 8 minutes. At the end of the simulation, participants were debriefed based on comparison of their performance with the action checklist.

### Main Measures

The main measures assessed were subject checklist completion rates, global rating scales (GRS), time to critical actions, and pre-post simulation confidence surveys.

### Instrument Development

There are no existing performance expectations for in-flight medical emergency responders that can serve as an external validity check. However, after literature review, consensus discussions among flight attendants (MC), aviation (MDS, CT, and PA), and emergency medicine experts (NN, WB) led to the development of optimal performance expectations reflected in scenario-specific rating forms, including both checklists and GRS. These were cross-checked for content validity by having other team members (MDS, RB) review the checklist items. Items included history gathering, physical examination, basic management choices, and communications actions.

The 4-point GRS (1=needs further instruction, 2=competent but with close supervision, 3=competent with minimal supervision, and 4=competent to perform independently) measures competence in managing the scenario and is similar to the entrustable professional activities scale used in undergraduate medical education [23]. We ran four pilot simulations per case with a sample group of resident physician participants. This allowed us to train the standardized participants and refine our simulation cases and checklist items. Some items were reworded for clarity, and several were dropped.

We also created pre-post simulation surveys for participants to self-assess their readiness for in-flight medical emergencies, knowledge of resources, medicolegal concerns, crew integration, in-flight medical emergency communications process, and willingness to respond. Surveys were pilot-tested for clarity, and usability questions (app group only) were derived from a previously developed technology usability survey [24] and administered immediately before and after the simulations.

### Observation, Rating Method, and Data Collection

Raters were physicians and nurses, with research expertise, who were trained on the checklists for the scenarios. Raters had no conflicts of interest. Participants in the app group often had the app in hand; thus, we could not blind the raters. Primary and secondary raters observed behind two-way glass in the control room. Due to scheduling logistics, secondary rating was occasionally performed using the audiovisual recording. Because this occurred in less than 5% of cases, we did not test interrater reliability between live and video ratings. All observations were captured on paper, transferred into survey software (Qualtrics), and then extracted to a spreadsheet program (Excel v2013, Microsoft Corp). Checklist items were marked as either “observed” or “not observed.” Something could be “not observed” if it was not done, time ran out, the standardized participant patient prevented the action, or the observer missed the action. Five actions per case were timed. The number of these items was limited due to rater burden. Recorded times of the two raters were averaged together for statistical analysis.

## Results

Sample size estimation was difficult due to unknown performance expectations, standard deviations, and effect sizes. However, we prospectively estimated our sample size to be 74 in total, or 37 per group, to have an 80% chance (power=0.80) of detecting a 20% improved performance overall in the checklist, with an assumed standard deviation of 30%. The study was stopped after interim analysis (29 participants per arm) due to resource constraints.

All statistical tests were performed against a two-sided alternative hypothesis with a significance level of 5% (α=.05) using R version 3.2.5 or latest version. Interrater reliability was calculated using Gwet’s AC1 (agreement coefficient 1), which is capable of handling more than two raters and response categories. The proportion of participants to complete each action, treated as binary variables, were compared using chi-square analysis or Fisher exact test as appropriate. In addition, the percentage of applicable completed actions was averaged between raters and compared between groups using independent sample *t* tests. The Likert-type global competency ratings and response times for the timed critical actions were not normally distributed, so both were compared using nonparametric Wilcoxon rank sum tests. Demographics were analyzed between groups using chi-square or Fisher exact test as appropriate. Mean ratings on the pre- and postsimulation surveys were analyzed using a linear mixed model. A log transformation was used as needed to meet model assumptions.

Analysis of participant demographics did not show differences in specialty, level of training, experience flying, or experience with in-flight medical emergencies (Table 1) between the two groups. The mean interrater reliability across the entire case was 0.90 for the syncope case and 0.94 for the shortness of breath case.

**Table 1:** Participant demographics (N=58)

Category		App (n=29), n (%)	Control (n=29), n (%)	*P* value^a^
**Specialty**				.75
	Internal medicine	8 (28)	8 (28)	
	Medicine-pediatrics	5 (17)	9 (31)	
	Pediatrics	4 (14)	3 (10)	
	Family medicine	1 (3)	2 (7)	
	Obstetrics and gynecology	2 (7)	2 (7)	
	Other (radiology and psychiatry)	9 (31)	5 (17)	
**Training level**				.84
	Postgraduate year 1	11 (38)	10 (35)	
	Postgraduate year 2	9 (31)	8 (28)	
	Postgraduate year 3	5 (17.2)	8 (28)	
	Postgraduate year 4	4 (13.8)	3 (10)	
**Direct high acuity care**				.54
	Rarely, if ever	2 (7)	4 (14)	
	Infrequently	6 (21)	6 (21)	
	Regularly, but not frequently	10 (35)	13 (45)	
	Frequently	8 (28)	3 (10)	
	Very frequently	3 (10)	3 (10)	
**Announcement for medical professionals**				.49
	Yes	6 (21)	4 (14)	
	No	23 (79)	25 (86)	
**Average number of flights per year**				.04
	None	0 (0)	2 (7)	
	1 to 2	11 (38)	14 (48)	
	3 to 5	15 (52)	6 (21)	
	6 to 10	3 (10)	7 (24)	
**Experienced call for medical help**				.19
	Once	1 (17)	3 (75)	
	2-3 times	5 (83)	1 (25)	
**Responded to call for medical help**				.99
	Never	3 (50)	2 (50)	
	Once	1 (17)	1 (25)	
	2-3 times	2 (33)	1 (25)	
**Actively provided care**				.99
	No, other provided care	1 (33)	0 (0)	
	Yes, I actively provided care	2 (67)	2 (100)	

^a^Used Fisher extract test for *P* values.

**Table 2:** Counts and proportion of completed checklist items (both raters).

Checklist item		Completed checklist item, n (%)		*P* value
		App (n=29)	Control (n=29)	
**Shortness of breath checklist items**				
	Introduces self and role	47 (81)	43 (74)	.37
	Acknowledges patient by name and identifies family members	45 (78)	18 (31)	<.001
	Asks patient for bystander for insight	58 (100)	57 (98)	.99
	Request flight attendant assistance	35 (60)	19 (33)	.003
	Informs/updates the cabin crew	17 (31)	5 (11)	.01
	Asks patient basic history	55 (95)	57 (98)	.62
	Asks patient allergies	17 (30)	17 (29)	.99
	Asks patient about home oxygen use	10 (17)	12 (21)	.64
	Elicits COPD^a^/asthma history	12 (21)	12 (21)	.99
	Examines heart and lungs through auscultation	46 (79)	54 (93)	.03
	Examines neck	2 (3)	2 (3)	.99
	Examines for pedal edema	6 (10)	8 (14)	.57
	Obtains vitals (BP^b^, HR^c^)	13 (22)	13 (22)	.99
	Reassesses patient	56 (97)	58 (100)	.50
	Administers steroids	18 (32)	4 (7)	.001
	Administers albuterol treatment	56 (97)	56 (97)	.99
	Requests for emergency medical kit	58 (100)	58 (100)	.99
	Requests ground medical control consult	26 (45)	11 (19)	.003
	Repeats vitals	38 (66)	52 (88)	.002
	Administers high flow oxygen	49 (85)	40 (69)	.048
**Syncope checklist items**				
	Introduces self and role	45 (78)	44 (79)	.90
	Acknowledges patient by name and identifies family members	27 (47)	18 (32)	.10
	Asks patient for bystander for insight	57 (98)	53 (91)	.21
	Requests emergency medical kit	58 (100)	58 (100)	.99
	Informs the cabin crew	39 (71)	18 (40)	.002
	Requests ground medical control	29 (50)	16 (28)	.02
	Asks about patient’s allergies	9 (15)	14 (25)	.23
	Asks about patient’s symptoms	54 (93)	57 (100)	.12
	Asks about palpitations	11 (19)	2 (4)	.01
	Asks about chest pain	33 (57)	27 (47)	.31
	Asks about dyspnea	35 (60)	22 (39)	.02
	Asks about arrhythmia history	15 (26)	11 (20)	.40
	Asks about gastrointestinal bleeding history	0 (0)	3 (5)	.12
	Asks patient basic history	58 (100)	54 (95)	.12
	Requests flight attendant assistance	24 (41)	5 (9)	<.001
	Auscultates heart and lungs	42 (72)	43 (74)	.83
	Examines abdomen	2 (3)	2 (3)	.99
	Examines patients neck	2 (3)	3 (5)	.99
	Requests the AED^d^	20 (35)	12 (21)	.12
	Obtains vitals	51 (88)	51 (88)	.99
	Repeats vitals	17 (29)	24 (41)	.17
	Reassess patient	56 (98)	52 (90)	.11
	Positions the patient	48 (84)	32 (56)	.001
	Administers oxygen	28 (52)	8 (14)	<.001
	Administers fluids	54 (93)	56 (97)	.68

^a^COPD: chronic obstructive pulmonary disease.

^b^BP: blood pressure.

^c^HR: heart rate/pulse.

^d^AED: automatic external defibrillator.

The app group had a significantly higher mean percentage of total completed checklist items (mean 58.0, SD 8.1) compared with the control group (mean 49.8, SD 7.0) for the syncope scenario (*t*_56_=4.15, *P*<.001) and the shortness of breath scenario (mean 56.1, SD 10.3 versus mean 49.4, SD 7.4 for control; *t*_56_=2.82, *P=*.007).

For both cases, the app group demonstrated significantly greater requests for ground medical control, flight attendant assistance, and communications to inform and update the cabin crew. For the shortness of breath case, the app demonstrated significantly greater administration of steroids, administration of high flow oxygen, and communications to inform and update the cabin crew. However, the control group completed the cardiac and pulmonary exams and reassessed vitals more frequently. For the syncope case, the app group asked about dyspnea and palpitations, positioned the patient supine, and administered oxygen more frequently compared with the control group (Table 2).

For timed actions, the app group had significantly shorter response times for the “alert ground medical support” checklist item compared with the control group, and this was statistically significant for both cases (*P*=.01; Table 3). However, the control group for the shortness of breath case had a statistically significant shorter response time for the “obtains vitals” checklist item (*P*=.006; Table 3).

**Figure 3:**
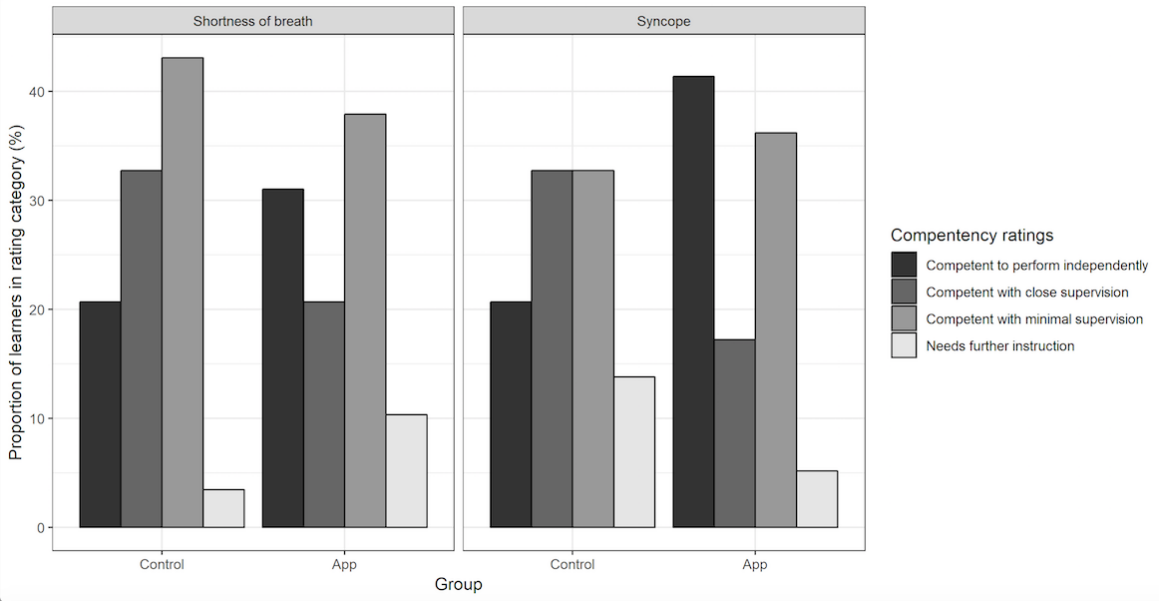
Global ratings for shortness of breath and syncope. IFME: in-flight medical emergency; SOB: shortness of breath; SYN: syncope.

Comparing the performance of learners across the two groups, there was no significant difference in the GRS for the shortness of breath case; however, the app group was rated significantly higher (mean 3.14, SD 0.89) for the syncope case compared with the control group (mean 2.6, SD 0.97; *P*=.003; Figure 3). Additionally, although not statistically significant, there seemed to be a trend with upper postgraduate levels performing slightly better, and certain specialties (internal medicine) performing better than other ones (radiology).

**Table 3:** Timed critical actions for shortness of breath and syncope cases.

Timed critical actions		App (n=29), mean (SD)	Control (n=29), mean (SD)	*P* value
**Shortness of breath**				
	Albuterol	264.6 (104.4)	239.9 (95.2)	.35
	Oxygen	268.0 (127.0)	294.0 (157.7)	.59
	Ground medical crew	369.8 (143.4)	452.2 (78.2)	.01
	Emergency medical kit	106.1 (35.5)	108.5 (36.0)^a^	.74
	Vitals	336.5 (125.9)	249.0 (113.6)^a^	.006
**Syncope**				
	Emergency medical kit	101.7 (40.5)	82.7 (33.5)	.05
	Ground medical crew	352.4 (155.0)	441.5 (87.0)	.02
	Vitals	265.2 (107.7)	228.2 (122.9)	.05
	Supine position	282.8 (139.5)	321.4 (167.8)^a^	.32
	Give fluids	314.1 (107.4)	260.3 (83.3)	.05

^a^n=28.

**Figure 4:**
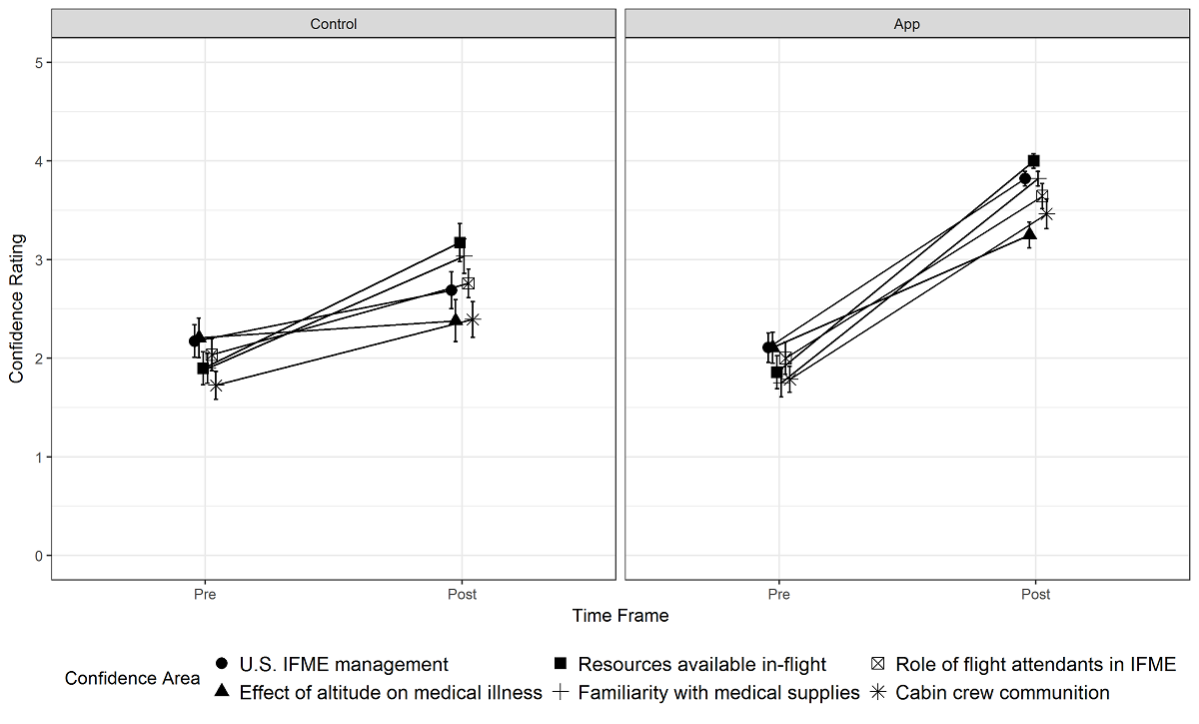
Pre- and postsimulation learner confidence in managing in-flight medical emergencies (IFME).

Postsimulation surveys (Figure 4) were associated with higher ratings from both the app and control groups relative to presurvey. However, the app group demonstrated increased confidence compared with the control group.

## Discussion

### Principal Findings

Availability of medical care during an in-flight medical emergency is a passenger safety prerogative. To address this need, most US airlines mandate first aid and basic life support training for flight crew and contact with ground-based medical consultation services, generally staffed by emergency medicine physicians who provide protocol-driven treatment recommendations and help make decisions regarding plane diversion. Health care professionals are not obligated to volunteer, and factors influencing their willingness to respond include their specialty, ancillary training (eg, combat medic, paramedic), years of practice, and medicolegal concerns [25]. Our survey showed that confidence increased with training in this unfamiliar environment, but confidence to respond increased more in the app group. This increase in confidence should not be falsely reassuring of anticipated improved performance, but the user may be more likely to respond.

We successfully simulated in-flight medical emergencies by recreating space constraints, communications barriers, and equipment limitations present on a mobile aircraft. We noted improved performance in actions where the app encouraged communication with flight crew and ground medical support. The app helps ensure that the proper questions are asked of patients, which may yield more fruitful conversations with ground medical control and improve treatments administered. Effective communications with cabin crew and ground medical control are crucial so that ground medical control can advise the pilots on the need to divert for care. Such decisions are costly and affect the passengers on many levels [26].

The app both offers a cognitive aid similar to an Advanced Cardiac Life Support card, while simultaneously introducing an additional source of cognitive load [27,28]. We believe that certain actions or times could be positively or negatively affected by the app. For example, vitals or certain history or physical exam items might be delayed while the learner was reading the app. Although some reached statistical significance (auscultation favored control in the shortness of breath group), there was not a clear preponderance favoring the control group for these types of actions. The app makes it clear that high flow oxygen is indicated due to altitude, and we noted improvements in that choice. Similarly, we saw improvements in the supine positioning for the app group in the syncope case, which is prompted by the app. Overall, the app group completed a higher percentage of checklist items compared with the control group in both cases. However, we should caution that the app could delay times to basic physical assessment, including vital signs.

The literature has shown that checklists and GRS can be complementary [29]. GRS are sometimes better able to see subtle signs of expertise than checklists; however, checklists give raters very concrete items to view and thus may improve interrater reliability. In our study, we found that GRS did not show improved performance with the app in the shortness of breath case but did in the syncope case. Possible reasons for this include rating effects, practice effect with app (syncope case was always second), and additional preparation with the app during the approximately 10-minute break between simulation cases.

### Limitations

Our limitations include the small amount of time learners interacted with the app before using it. We gave participants 15 minutes to familiarize themselves with the app, and chose this given average taxi-out times of 16.2 minutes [30]. Our simulation scenarios were relatively short at 8 minutes each, and extra time might have given either group a chance to meet missed checklist items. We did not control for the confounding variable of other app usage, and although we did not formally track this, we noted very little alternate health care app use. It is difficult to know how actual real-world performance would progress, but our gestalt was that the environment, cases, and witnessed performance were quite credible. Our relayed via cabin crew communication method for ground medical support was held constant and mimicked that found on many airlines, but there is no standard expectation. We expect changing technology and situational urgency will alter the method of communication. We also did not analyze for standardized participant effects, but we had nearly the same cohort throughout the entire project. We did not blind the raters because it was clear due to the app use in view. In hindsight, we could have given both groups the same device to create partial blinding. We anticipated a case order effect and we kept our case order the same for this reason. We did not take a G-theory approach to looking at the variability in case, case order, standardized participants, and raters, in part because sample size would have been prohibitively large. Our results are likely also subject to volunteer bias in that those who were more trained, able, and confident to perform likely self-volunteered for our study. Finally, our study focused on resident physicians; however, the electronic app being tested is also applicable to allied health professions or physicians some years out of residency training, which would make for an interesting future study.

### Conclusion

We found that the use of a mobile phone app modestly improved performance of nonemergency resident physician participants during simulated in-flight medical emergencies. We caution that app use may delay or distract from basic physical assessments. The app improved participants’ confidence in in-flight medical emergency response more than simulation practice alone.

### Future Directions

Future studies are needed to examine whether the app is used during real in-flight medical emergencies. It will also be interesting to examine the effect of introducing this electronic app to other health care professionals as well as attending physicians. Finally, we maintained the contents of our airline emergency kits as per Federal Aviation Administration guidelines; however, international flights may have considerable variations in medical kit contents. It might be useful to investigate the effects of different medical kits on the management of simulated in-flight medical emergencies.

## Appendix

Multimedia Appendix 1 Overview of the airRx app.

Multimedia Appendix 2 CONSORT‐EHEALTH checklist (V 1.6.1).

## Abbreviations

AED: automatic external defibrillator
BP: blood pressure
COPD: chronic obstructive pulmonary disease
GRS: global rating scale
HR: heart rate/pulse
JITT: just-in-time training
