# Supplementary figures and images for: Impact of an Electronic App on Resident Responses to Simulated In-Flight Medical Emergencies: Randomized Controlled Trial
Source: JMIR Med Educ. 2019 Jun 7;5(1):e10955. doi: 10.2196/10955 (PMC6594212; doi:10.2196/10955)

## Slide 1
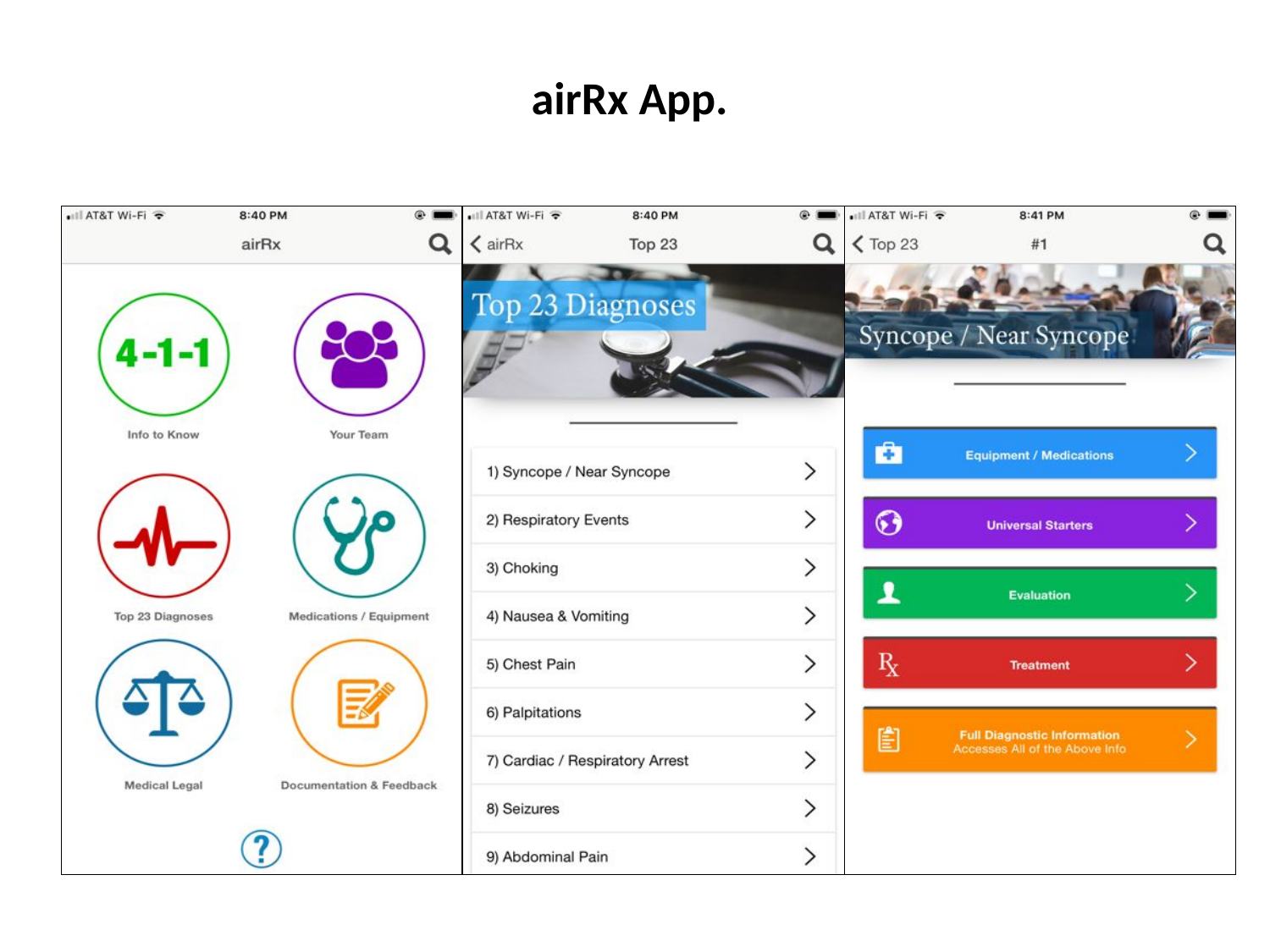

# airRx App.

Supplement: Multimedia Appendix 1 [file mededu_v5i1e10955_app1.pptx]
