# Supplementary material for: Impact of an Electronic App on Resident Responses to Simulated In-Flight Medical Emergencies: Randomized Controlled Trial
Source: JMIR Med Educ. 2019 Jun 7;5(1):e10955. doi: 10.2196/10955 (PMC6594212; doi:10.2196/10955)
Supplement: Multimedia Appendix 2 [file mededu_v5i1e10955_app2.pdf]

CONSORT-EHEALTH Checklist V1.6.2 Report	Manuscript Number	10955
**(based on CONSORT-EHEALTH V1.6), available at [http://tinyurl.com/consort-ehealth-v1-6].**		
**Date completed**		
6/4/2019 17:59:25		
**by**		
Nur-Ain Nadir		
A randomized control trial of the impact of an electronic application on resident responses to simulated in-flight medical emergencies		
TITLE		
**1a-i) Identify the mode of delivery in the title**		
Yes" " A randomized control trial of the impact of an electronic application on resident responses to simulated in-flight medical emergencies"		
**1a-ii) Non-web-based components or important co-interventions in title**		
Yes: " This was a randomized study of volunteer, non-emergency resident physician subjects who managed simulated IFMEs with or without the app. "		
**1a-iii) Primary condition or target group in the title**		
Yes: "This was a randomized study of volunteer, non-emergency resident physician subjects who managed simulated IFMEs with or without the app."		
ABSTRACT		
**1b-i) Key features/functionalities/components of the intervention and comparator in the METHODS section of the ABSTRACT**		
No. This study was not focusing on the functionality aspects of the app.		
**1b-ii) Level of human involvement in the METHODS section of the ABSTRACT**		
**1b-iii) Open vs. closed, web-based (self-assessment) vs. face-to-face assessments in the METHODS section of the ABSTRACT**		
Not applicable to this study		
**1b-iv) RESULTS section in abstract must contain use data**		
Yes.		
**1b-v) CONCLUSIONS/DISCUSSION in abstract for negative trials**		
Not applicable to this study		
INTRODUCTION		
**2a-i) Problem and the type of system/solution**		

Yes.<br>" Epidemiologic evidence for in-flight medical emergencies (IFMEs) from a ground-based medical support system estimated that medical emergencies occur in 1 of every 604 flights [1]. This is likely an underestimate, because no mandatory reporting system exists and uncomplicated issues often go unreported [2]. Air travel is increasing, with 895.5 million passengers flying in 2015 [3], leading to increased frequency of IFMEs. In one study, 42% of 418 healthcare providers surveyed reported being called upon to give aid in an IFME [4].<br>The Federal Aviation Administration (FAA) mandates that United States–based airlines carry basic first-aid kits stocked with bandages and splints, and at least one automated external defibrillator (AED) must be available [5]. Beyond the basic kit, no national or international standards exist, though there have been recent calls for consistency [6,7].<br>Healthcare personnel are also unlikely to be familiar with medical kit contents, flight crew communication and medical emergency protocols [4]. Clinicians' expertise typically consists of their specialty training and life support courses. Emergency response training is often limited as emergency medicine is not a mandatory rotation in medical education [8]. Though helpful, ground- based medical consultation support services (ground medical control) still depend on volunteers to be their “eyes and ears” [9,10]. The assumption is that volunteers will find and report clinical information relevant to the presenting medical emergency [10].<br>Comfort attending to an IFME is likely to vary substantially across provider backgrounds. Thus, there is a need for education about the environment, and scenario-based basic IFME response training. In recent months, the aviation and healthcare industries have recognized this and called for education in emergency stabilization and flight medicine at both graduate and undergraduate levels [12,13].<br>Although several authors have discussed the management of in-flight emergencies [14-19], little real-time decision support exists outside of ground medical control. Normal emergency response smartphone applications (apps) or cognitive aids may not take the environment into account. In response to this perceived need, a smartphone app was designed by emergency, aerospace medicine, and radiology physicians (airRx) [20] to assist licensed healthcare personnel in dealing with the most common IFMEs. The app offers complaint-specific recommended actions, care algorithms, and in-the-moment information regarding the likely available medications. While serving as a real-time decision support reference, the app also provides a method of just-in-time-training (JITT) [21]. Pertinently, the JITT approach has been successful in on-the-job training for first responders in unfamiliar situations [22]. Studies have also shown that smartphone based cognitive aids promote adherence to protocols in both real and simulated clinical scenarios [23–25]. A JITT-based smart phone cognitive aid/application is therefore, a reasonable approach to delivering focused learning during an IFME. The objective of this study was to determine usefulness of the airRx smartphone application in responding to simulated IFMEs. Our secondary objective was to examine whether access to the airRx app would increase confidence to respond to an IFME."		
**2a-ii) Scientific background, rationale: What is known about the (type of) system**		

Yes:<br>"Epidemiologic evidence for in-flight medical emergencies (IFMEs) from a ground-based medical support system estimated that medical emergencies occur in 1 of every 604 flights [1]. This is likely an underestimate, because no mandatory reporting system exists and uncomplicated issues often go unreported [2]. Air travel is increasing, with 895.5 million passengers flying in 2015 [3], leading to increased frequency of IFMEs. In one study, 42% of 418 healthcare providers surveyed reported being called upon to give aid in an IFME [4].<br>The Federal Aviation Administration (FAA) mandates that United States–based airlines carry basic first-aid kits stocked with bandages and splints, and at least one automated external defibrillator (AED) must be available [5]. Beyond the basic kit, no national or international standards exist, though there have been recent calls for consistency [6,7].<br>Healthcare personnel are also unlikely to be familiar with medical kit contents, flight crew communication and medical emergency protocols [4]. Clinicians' expertise typically consists of their specialty training and life support courses. Emergency response training is often limited as emergency medicine is not a mandatory rotation in medical education [8]. Though helpful, ground- based medical consultation support services (ground medical control) still depend on volunteers to be their "eyes and ears" [9,10]. The assumption is that volunteers will find and report clinical information relevant to the presenting medical emergency [10].<br>Comfort attending to an IFME is likely to vary substantially across provider backgrounds. Thus, there is a need for education about the environment, and scenario-based basic IFME response training. In recent months, the aviation and healthcare industries have recognized this and called for education in emergency stabilization and flight medicine at both graduate and undergraduate levels [12,13].<br>Although several authors have discussed the management of in-flight emergencies [14-19], little real-time decision support exists outside of ground medical control. Normal emergency response smartphone applications (apps) or cognitive aids may not take the environment into account. In response to this perceived need, a smartphone app was designed by emergency, aerospace medicine, and radiology physicians (airRx) [20] to assist licensed healthcare personnel in dealing with the most common IFMEs. The app offers complaint-specific recommended actions, care algorithms, and in-the-moment information regarding the likely available medications. While serving as a real-time decision support reference, the app also provides a method of just-in-time-training (JITT) [21]. Pertinently, the JITT approach has been successful in on-the-job training for first responders in unfamiliar situations [22]. Studies have also shown that smartphone based cognitive aids promote adherence to protocols in both real and simulated clinical scenarios [23–25]. A JITT-based smart phone cognitive aid/application is therefore, a reasonable approach to delivering focused learning during an IFME. The objective of this study was to determine usefulness of the airRx smartphone application in responding to simulated IFMEs. Our secondary objective was to examine whether access to the airRx app would increase confidence to respond to an IFME."		
**Does your paper address CONSORT subitem 2b?**		

Yes: "Epidemiologic evidence for in-flight medical emergencies (IFMEs) from a ground-based medical support system estimated that medical emergencies occur in 1 of every 604 flights [1]. This is likely an underestimate, because no mandatory reporting system exists and uncomplicated issues often go unreported [2]. Air travel is increasing, with 895.5 million passengers flying in 2015 [3], leading to increased frequency of IFMEs. In one study, 42% of 418 healthcare providers surveyed reported being called upon to give aid in an IFME [4].<br>The Federal Aviation Administration (FAA) mandates that United States-based airlines carry basic first-aid kits stocked with bandages and splints, and at least one automated external defibrillator (AED) must be available [5]. Beyond the basic kit, no national or international standards exist, though there have been recent calls for consistency [6,7].<br>Healthcare personnel are also unlikely to be familiar with medical kit contents, flight crew communication and medical emergency protocols [4]. Clinicians' expertise typically consists of their specialty training and life support courses. Emergency response training is often limited as emergency medicine is not a mandatory rotation in medical education [8]. Though helpful, ground- based medical consultation support services (ground medical control) still depend on volunteers to be their "eyes and ears" [9,10]. The assumption is that volunteers will find and report clinical information relevant to the presenting medical emergency [10].<br>Comfort attending to an IFME is likely to vary substantially across provider backgrounds. Thus, there is a need for education about the environment, and scenario-based basic IFME response training. In recent months, the aviation and healthcare industries have recognized this and called for education in emergency stabilization and flight medicine at both graduate and undergraduate levels [12,13].<br>Although several authors have discussed the management of in-flight emergencies [14-19], little real-time decision support exists outside of ground medical control. Normal emergency response smartphone applications (apps) or cognitive aids may not take the environment into account. In response to this perceived need, a smartphone app was designed by emergency, aerospace medicine, and radiology physicians (airRx) [20] to assist licensed healthcare personnel in dealing with the most common IFMEs. The app offers complaint-specific recommended actions, care algorithms, and in-the-moment information regarding the likely available medications. While serving as a real-time decision support reference, the app also provides a method of just-in-time-training (JITT) [21]. Pertinently, the JITT approach has been successful in on-the-job training for first responders in unfamiliar situations [22]. Studies have also shown that smartphone based cognitive aids promote adherence to protocols in both real and simulated clinical scenarios [23-25]. A JITT-based smart phone cognitive aid/application is therefore, a reasonable approach to delivering focused learning during an IFME. The objective of this study was to determine usefulness of the airRx smartphone application in responding to simulated IFMEs. Our secondary objective was to examine whether access to the airRx app would increase confidence to respond to an IFME.<br>"		
METHODS		
**3a) CONSORT: Description of trial design (such as parallel, factorial) including allocation ratio**		
Yes: "This was a prospective randomized control trial. Fifty-eight subjects were block randomized by post-graduate year and specialty area to simulated IFMEs with and without access to a smart device application."		
**3b) CONSORT: Important changes to methods after trial commencement (such as eligibility criteria), with reasons**		
No changes to methods were made.		
**3b-i) Bug fixes, Downtimes, Content Changes**		
Not applicable		
**4a) CONSORT: Eligibility criteria for participants**		
"Subjects were solicited from non-emergency medicine residency programs including Diagnostic Radiology, Family Medicine, Internal Medicine, Pediatrics, Psychiatry, Combined Medicine-Pediatrics, and Obstetrics and Gynecology. Emergency medicine residents were excluded given their expertise and training in management of emergencies. Subjects' performances were kept confidential. They were compensated through a $25 gift card and a copy of the airRx application at no cost to them. Subjects were instructed to keep the scenarios confidential to minimize the relay of scenario information to future participants. "		
**4a-i) Computer / Internet literacy**		

No this was not deemed relevant for this study		
**4a-ii) Open vs. closed, web-based vs. face-to-face assessments:**		
Yes:<br><br>"Subjects were solicited from non-emergency medicine residency programs including Diagnostic Radiology, Family Medicine, Internal Medicine, Pediatrics, Psychiatry, Combined Medicine-Pediatrics, and Obstetrics and Gynecology. Emergency medicine residents were excluded given their expertise and training in management of emergencies. Subjects' performances were kept confidential. They were compensated through a $25 gift card and a copy of the airRx application at no cost to them. Subjects were instructed to keep the scenarios confidential to minimize the relay of scenario information to future participants. "		
**4a-iii) Information giving during recruitment**		
Yes.<br>"All subjects were pre-briefed via a standardized script. Both the control and intervention groups were allowed to use any other phone apps they had on their personal smart device that would be accessible during airplane mode. The application group had up to 15 minutes to familiarize themselves with the app. Both groups were aware that the simulation topic was IFME. Scenarios began with the subjects sitting in the simulated cabin with a brief pause before flight attendants announced the IFME and called for assistance. "		
**4b) CONSORT: Settings and locations where the data were collected**		
Yes: "The study took place at a university hospital affiliated simulation center. Space and movement limits that mimicked the floor distances of a Boeing 737 aircraft were created within a simulation lab with audiovisual recording capability. "		
**4b-i) Report if outcomes were (self-)assessed through online questionnaires**		
Yes."We also created pre-post simulation surveys for participants to self-assess their readiness for IFMEs, knowledge of resources, medico-legal concerns, crew integration, IFME communications process, and willingness to respond. Surveys were pilot tested for clarity, and usability questions (app group only) were derived from a previously developed technology usability survey"		
**4b-ii) Report how institutional affiliations are displayed**		
Not relevant		
**5) CONSORT: Describe the interventions for each group with sufficient details to allow replication, including how and when they were actually administered**		
**5-i) Mention names, credential, affiliations of the developers, sponsors, and owners**		
Yes.		
**5-ii) Describe the history/development process**		
This was not relevant to this study.		
**5-iii) Revisions and updating**		
Not applicable		
**5-iv) Quality assurance methods**		
Not applicable to this study		
**5-v) Ensure replicability by publishing the source code, and/or providing screenshots/screen-capture video, and/or providing flowcharts of the algorithms used**		
Not applicable		
**5-vi) Digital preservation**		
Not applicable		
**5-vii) Access**		
Yes: "All subjects were pre-briefed via a standardized script. Both the control and intervention groups were allowed to use any other phone apps they had on their personal smart device that would be accessible during airplane mode. The application group had up to 15 minutes to familiarize themselves with the app. Both groups were aware that the simulation topic was IFME"		
**5-viii) Mode of delivery, features/functionalities/components of the intervention and comparator, and the theoretical framework**		

This item was not deemed relevant. The App was not created by authors, they merely tested its utility		
**5-ix) Describe use parameters**		
NOt important		
**5-x) Clarify the level of human involvement**		
The App is meant to be used for Inflight Medical emergencies. "The simulation center has a cadre of standardized participants (SPs) who undergo general and scenario-specific orientation. The SPs went through dry runs of each scenario, received feedback on their performance, and were given ear buds for prompts in real-time. In each scenario there was one SP passenger who became ill and one SP passenger bystander who had relevant information if asked. Stable actor cohorts played these roles. Pathologic physical exam findings were given on cue through pre-written cards from the bystander SP, as healthy patient SPs could not mimic symptoms such as wheezing.<br>For each case there were also two SP flight attendants who communicated with the investigators in the simulation control room ("pilot" and "ground medical support"), and relayed responses to the participants. Real flight attendants trained SPs to portray flight attendant roles through direct observations of their performance in pilot simulations, video review, and discussion of planned responses to questions. In order to isolate subject performances, we instructed the flight attendants to be helpful and follow directions, but wait to inform ground medical control until instructed. Thus, we controlled for variable airline protocols, flight attendant training, and individual responses expected in real life.<br>"		
**5-xi) Report any prompts/reminders used**		
No prompts were used to elicit subjects to use the app.		
**5-xii) Describe any co-interventions (incl. training/support)**		
Not applicable to this study.		
**6a) CONSORT: Completely defined pre-specified primary and secondary outcome measures, including how and when they were assessed**		
Yes. "The main measures assessed were subject CL completion rates, GRS, time to critical actions, and pre-post simulation confidence surveys. "		
**6a-i) Online questionnaires: describe if they were validated for online use and apply CHERRIES items to describe how the questionnaires were designed/deployed**		
Not applicable to this study.		
**6a-ii) Describe whether and how "use" (including intensity of use/dosage) was defined/measured/monitored**		
not applicable		
**6a-iii) Describe whether, how, and when qualitative feedback from participants was obtained**		
not applicable		
**6b) CONSORT: Any changes to trial outcomes after the trial commenced, with reasons**		
Yes: "The study took place at a university hospital affiliated simulation center. Space and movement limits that mimicked the floor distances of a Boeing 737 aircraft were created within a simulation lab with audiovisual recording capability. "		
**7a) CONSORT: How sample size was determined**		
**7a-i) Describe whether and how expected attrition was taken into account when calculating the sample size**		
"Sample size estimation was difficult due to unknown performance expectations, standard deviations,<br>and effect sizes. However, we prospectively estimated our sample size to be 74 total, or 37 per group, to have an 80% chance (power = 0.80) of detecting a 20% improved performance overall in the CL, with an assumed standard deviation of 30%."		
**7b) CONSORT: When applicable, explanation of any interim analyses and stopping guidelines**		
Yes. "The main measures assessed were subject CL completion rates, GRS, time to critical actions, and pre-post simulation confidence surveys. "		
**8a) CONSORT: Method used to generate the random allocation sequence**		
"Fifty-eight subjects were block randomized by post-graduate year and specialty area to simulated IFMEs with and without access to a smart device application. "		
**8b) CONSORT: Type of randomisation; details of any restriction (such as blocking and block size)**		
"Fifty-eight subjects were block randomized by post-graduate year and specialty area to simulated IFMEs with and without access to a smart device application. "		

**9) CONSORT: Mechanism used to implement the random allocation sequence (such as sequentially numbered containers), describing any steps taken to conceal the sequence until interventions were assigned**		
Block randomization was performed through pulling names from a container but has not been explained in detail in the paper.		
**10) CONSORT: Who generated the random allocation sequence, who enrolled participants, and who assigned participants to interventions**		
Research coordinator who was blinded to participants other than their initials and PGY levels		
**11a) CONSORT: Blinding - If done, who was blinded after assignment to interventions (for example, participants, care providers, those assessing outcomes) and how**		
**11a-i) Specify who was blinded, and who wasn't**		
Only the research coordinator who was in charge of block randomization was blinded but not the actual evaluators who were evaluating the intervention.		
**11a-ii) Discuss e.g., whether participants knew which intervention was the "intervention of interest" and which one was the "comparator"**		
Yes. participants were aware of this.		
**11b) CONSORT: If relevant, description of the similarity of interventions**		
not applicable		
**12a) CONSORT: Statistical methods used to compare groups for primary and secondary outcomes**		
"All statistical tests were performed against a two-sided alternative hypothesis with a significance level of 5% (a = 0.05) using R version 3.2.5 or latest version. Inter-rater reliability was calculated using Gwet's AC1 (Agreement Coefficient 1) which is capable of handling more than two raters and response categories. The proportion of participants to complete each action, treated as binary variables, were compared using chi-square analysis or Fisher's exact test as appropriate. In addition, the percentage of applicable completed actions was averaged between raters and compared between groups using independent sample t-tests. The Likert type global competency ratings and response times for the timed critical actions were not normally distributed, so both were compared using non-parametric Wilcoxon rank sum tests. Demographics were analyzed between groups using chi-square or Fisher's exact test as appropriate. Mean ratings on the pre and post simulation surveys were analyzed using linear mixed model. A log transformation was used as needed to meet model assumptions. "		
**12a-i) Imputation techniques to deal with attrition / missing values**		
not applicable to this study		
**12b) CONSORT: Methods for additional analyses, such as subgroup analyses and adjusted analyses**		
Not applicable to this stuyd.		
RESULTS		
**13a) CONSORT: For each group, the numbers of participants who were randomly assigned, received intended treatment, and were analysed for the primary outcome**		
27 per group.		
**13b) CONSORT: For each group, losses and exclusions after randomisation, together with reasons**		
Not applicable to this study		
**13b-i) Attrition diagram**		
Not applicable		
**14a) CONSORT: Dates defining the periods of recruitment and follow-up**		
Not applicable.		
**14a-i) Indicate if critical "secular events" fell into the study period**		
Not applicable		
**14b) CONSORT: Why the trial ended or was stopped (early)**		
Yes. "The study was stopped after interim analysis (29 subjects per arm) due to resource constraints. "		
**15) CONSORT: A table showing baseline demographic and clinical characteristics for each group**		
Included in paper.		
**15-i) Report demographics associated with digital divide issues**		

NOt applicable		
**16a) CONSORT: For each group, number of participants (denominator) included in each analysis and whether the analysis was by original assigned groups**		
**16-i) Report multiple “denominators” and provide definitions**		
Not applicable		
**16-ii) Primary analysis should be intent-to-treat**		
Not applicable		
**17a) CONSORT: For each primary and secondary outcome, results for each group, and the estimated effect size and its precision (such as 95% confidence interval)**		
Not applicable		
**17a-i) Presentation of process outcomes such as metrics of use and intensity of use**		
Not applicable		
**17b) CONSORT: For binary outcomes, presentation of both absolute and relative effect sizes is recommended**		
Not applicable		
**18) CONSORT: Results of any other analyses performed, including subgroup analyses and adjusted analyses, distinguishing pre-specified from exploratory**		
NOt applicable		
**18-i) Subgroup analysis of comparing only users**		
Not applicable		
**19) CONSORT: All important harms or unintended effects in each group**		
Not applicable		
**19-i) Include privacy breaches, technical problems**		
Not applicable		
**19-ii) Include qualitative feedback from participants or observations from staff/researchers**		
NOt applicable		
DISCUSSION		
**20) CONSORT: Trial limitations, addressing sources of potential bias, imprecision, multiplicity of analyses**		
**20-i) Typical limitations in ehealth trials**		
"Our limitations include the small amount of time learners interacted with the app prior to using it. We gave subjects 15 minutes to familiarize themselves with the app, and chose this given average taxi-out times of 16.2 minutes [31]. Our simulation scenarios were relatively short at 8 minutes each (arrived at through pilot testing), and extra time might have given either group a chance to meet missed CL items. We did not control for the confounding variable of other app usage, and while we did not formally track this, we noted very little alternate healthcare app use. While it is difficult to know how actual real world performance would progress, our gestalt was that the environment, cases, and witnessed performance were quite credible. Our relayed via cabin crew communication method for ground medical support was held constant and mimics that found on many airlines, but there is no standard expectation. We expect changing technology and situational urgency will alter the method of communication. We also did not analyze for standardized participant effects, but we had nearly the same cohort throughout the entire project. We did not blind the raters as it was clear due to the application use in view. In hindsight, we could have given both groups the same device to create partial blinding. We anticipated a case order effect and we kept our case order the same for this reason. We did not take a G-theory approach to looking at the variability in case, case order, standardized participants, and raters, in part because sample size would have been prohibitively large. "		
**21) CONSORT: Generalisability (external validity, applicability) of the trial findings**		
**21-i) Generalizability to other populations**		

"Our limitations include the small amount of time learners interacted with the app prior to using it. We gave subjects 15 minutes to familiarize themselves with the app, and chose this given average taxi-out times of 16.2 minutes [31]. Our simulation scenarios were relatively short at 8 minutes each (arrived at through pilot testing), and extra time might have given either group a chance to meet missed CL items. We did not control for the confounding variable of other app usage, and while we did not formally track this, we noted very little alternate healthcare app use. While it is difficult to know how actual real world performance would progress, our gestalt was that the environment, cases, and witnessed performance were quite credible. Our relayed via cabin crew communication method for ground medical support was held constant and mimics that found on many airlines, but there is no standard expectation. We expect changing technology and situational urgency will alter the method of communication. We also did not analyze for standardized participant effects, but we had nearly the same cohort throughout the entire project. We did not blind the raters as it was clear due to the application use in view. In hindsight, we could have given both groups the same device to create partial blinding. We anticipated a case order effect and we kept our case order the same for this reason. We did not take a G-theory approach to looking at the variability in case, case order, standardized participants, and raters, in part because sample size would have been prohibitively large. "		
**21-ii) Discuss if there were elements in the RCT that would be different in a routine application setting**		
not applicable		
**22) CONSORT: Interpretation consistent with results, balancing benefits and harms, and considering other relevant evidence**		
**22-i) Restate study questions and summarize the answers suggested by the data, starting with primary outcomes and process outcomes (use)**		
"We successfully simulated in flight medical emergencies (IFME) by recreating space constraints, communications barriers, and equipment limitations present on a mobile aircraft. We noted improved performance in actions where the app encouraged communication with flight crew and ground medical support. The app helps ensure that the proper questions are asked of patients, which may yield more fruitful conversations with ground medical control, and improve treatments administered. "		
**22-ii) Highlight unanswered new questions, suggest future research**		
Not applicable		
Other information		
**23) CONSORT: Registration number and name of trial registry**		
not applicable		
**24) CONSORT: Where the full trial protocol can be accessed, if available**		
not applicable		
**25) CONSORT: Sources of funding and other support (such as supply of drugs), role of funders**		
"This work was conducted at Jump Simulation, a collaboration of OSF HealthCare and the University of Illinois College of Medicine at Peoria. The Illinois Corporation, AirRx, is a non-profit foundation with 501-c3 status. AirRx owns the app, airRx, and supported the research by providing standardized patient meals and gift cards to participating subjects. AirRx also received funds from the period of time where it was selling the app, which partially defrayed the costs of app development. The airRx app has subsequently become free on both Android and iOS platforms."		
**X26-i) Comment on ethics committee approval**		
"This study design was approved by the local Institutional Review Board. "		
**x26-ii) Outline informed consent procedures**		
Included within the paper.		
**X26-iii) Safety and security procedures**		
not applicable		
**X27-i) State the relation of the study team towards the system being evaluated**		
"This work was conducted at Jump Simulation, a collaboration of OSF HealthCare and the University of Illinois College of Medicine at Peoria. The Illinois Corporation, AirRx, is a non-profit foundation with 501-c3 status. AirRx owns the app, airRx, and supported the research by providing standardized patient meals and gift cards to participating subjects. AirRx also received funds from the period of time where it was selling the app, which partially defrayed the costs of app development. The airRx app has subsequently become free on both Android and iOS platforms."		
